# Early-Onset Paternal Smoking and Offspring Adiposity: Further Investigation of a Potential Intergenerational Effect Using the HUNT Study

**DOI:** 10.1371/journal.pone.0166952

**Published:** 2016-12-02

**Authors:** David Carslake, Pia R. Pinger, Pål Romundstad, George Davey Smith

**Affiliations:** 1 MRC Integrative Epidemiology Unit at the University of Bristol, Bristol, United Kingdom; 2 School of Social and Community Medicine, University of Bristol, Bristol, United Kingdom; 3 Department of Economics, University of Bonn, Bonn, Germany; 4 Department of Public Health and General Practice, Norwegian University of Science and Technology, Trondheim, Norway; Hunter College, UNITED STATES

## Abstract

Recently it has been suggested that rearing conditions during preadolescence in one generation may affect health outcomes in subsequent generations. Such parental effects, potentially induced by epigenetic modifications in the germ line, have attracted considerable attention because of their implications for public health and social policies. Yet, to date, evidence in humans has been rare due to data limitations and much further investigation in large studies is required. The aim of this paper is to reproduce and extend a recent study which found that paternal smoking before age 11 was associated with elevated body mass index (BMI) among male offspring in the Avon Longitudinal Study of Parents and Children (ALSPAC). Using the Nord-Trøndelag Health (HUNT) Study, we find that paternal smoking during pre-adolescence (<age 11) is not reliably or strongly associated with BMI among sons, with an estimated association close to zero (mean difference in kg m^-2^ (95% CI) was -0.18 (-1.75, 1.39) for sons aged 12–19 and 0.22 (-0.53, 0.97) for all ages). Among daughters, early-onset paternal smoking was imprecisely associated with an elevated BMI (mean difference was 1.50 (0.00, 3.00) for daughters aged 12–19 and 0.97 (0.06, 1.87) for all ages). Our results do not support a son-specific association of the magnitude reported in the ALSPAC study and we consider it improbable that early onset paternal smoking should influence specifically sons' BMI in one population and daughters' BMI in another. However, despite our considerable sample size (>45,000 offspring), we cannot rule out a weaker association, perhaps common to sons and daughters, which would be consistent with the ALSPAC study. Alternatively, we discuss whether confounding, chance in parallel tests, or sample selection effects might explain the observed associations of early paternal smoking with offspring BMI.

## Introduction

There has been much recent interest in parental effects whereby adverse exposures to nutrition, behaviours and life circumstance in one generation transmit by means of epigenetic modifications to subsequent generations. Along these lines, a recent series of papers have suggested that food supply and smoking during male preadolescence might be associated with offspring longevity, health outcomes and obesity [1] [2] [3] [4]. The present paper reproduces and extends one of these recent studies [5] in which paternal smoking before the age of 11 years was associated with raised BMI, fat mass and waist circumference in sons but not daughters. Based on the Avon Longitudinal Study of Parents and Children (ALSPAC) questionnaire data on smoking behaviours of around 9900 fathers, 166 of which reported regular smoking before age 11, the authors of [5] found positive mean differences in son's BMI, waist circumference and fat mass with paternal smoking onset before age 11, which increased with the son's age from 7 to 17 years. The results have been interpreted as suggestive evidence of an environmentally-triggered biological effect response. The idea is that the father’s germ cells are exposed to cigarette smoke which then translates into different offspring phenotypes by means of epigenetic modifications [6] [7] [8] [9] [10] [11].

If increases in BMI among the next generation were indeed triggered by male exposures to toxic substances during preadolescence, this would have very important implications for public policy. In particular, such findings might contribute to explaining why the so-called obesity epidemic followed shortly after the smoking epidemic. However, data-wise, most such analyses are subject to a number of potential problems. First, data rarely contain truly exogenous variation in first generation adolescent health, i.e., variation in health that is uncorrelated with unobserved variables that also affect the outcome of interest. Instead, parental conditions such as smoking behaviours tend to be endogenously related to unobserved characteristics that might influence offspring health in other ways. Therefore, such observational associations have to be interpreted with caution. At the minimum, the sensitivity of such findings with respect to different sets of control variables (i.e., adjusting the underlying models for different sets of potential confounders) should be assessed. Invariance of the estimated effect to different settings with different confounding structures is then a necessary (but not a sufficient) condition for the existence of a causal biological relationship. Second, most studies consider a considerable number of associations between several exposure variables and/or several outcomes in several subsamples, increasing the risk of getting one or more false positive results. Third, samples on which these analyses can be conducted are often small and diverse in terms of exposure incidence rates, such that robust associations which are apparent in one dataset might prove unimportant in other settings. Replication studies are needed that investigate the external validation of such findings in large samples.

As a consequence, we initiated the current study on the effects of paternal smoking during pre-adolescence using the Nord-Trøndelag Health (HUNT) Study to re-investigate whether any patterns can be found in the data that hint towards a potential effect of paternal smoking onset during preadolescence on offspring BMI. Our goal was to provide external validation of the ALSPAC study [5], which found the onset of paternal smoking before puberty to be associated with higher BMI among sons but not daughters. In line with this prior study [5] we focus on the time period of < 11 years for paternal smoking onset. We report adjusted and unadjusted BMI results for sons and daughters aged up to 76.

## Materials and Methods

### Study population and data processing

The HUNT Study (see website: https://www.ntnu.edu/hunt) is a large health study conducted in the rural Norwegian county of Nord-Trøndelag. At each of three phases (HUNT1, 1984–1986; HUNT2, 1995–1997 and HUNT3, 2006–2008), every resident aged 20 or more was invited to participate and participation rates were 89, 70 and 54% of the eligible population in HUNT1, HUNT2 and HUNT3, respectively [12]. In addition, children aged 13–19 were invited to participate in partner studies (YoungHUNT1, 1995–1997; YoungHUNT2, 2000–2001 and YoungHUNT3, 2006–2007). HUNT and YoungHUNT (YH) participants completed health questionnaires including questions concerning their current smoking habits, age of smoking uptake and/or quitting, drinking habits, physical activity, employment status and level of education. Participants in HUNT1 and HUNT3 were asked if they had been intoxicated in the previous two or four weeks, respectively. Participants in YoungHUNT were asked how frequently they had seen their parents intoxicated. Participants also attended a clinic where, among other measurements, their height, weight and blood pressure were recorded. Linkage with national birth records identified family associations among HUNT and YoungHUNT participants. An initial extraction of all participating individuals with at least one participating parent yielded 66,246 offspring, approximately 53% of all HUNT participants. This study was approved by the Regional Committee for Medical Research Ethics central Norway—2010/69, REK midt. Each participant and the parents/legal guardians of participants younger than 16 years old gave their written consent to participate.

Where offspring participated in more than one round of HUNT or YOUNGHUNT, we took BMI data from the earliest available round, giving a mean offspring age of 29.1 years (range 12.1–76.0). Data for all other variables pertaining to the offspring were taken from the same round if possible. If they were missing in that round, the earliest recorded value was used. There was some evidence that recalled ages of smoking onset varied and tended to get older over time, i.e., as smoking became more stigmatized in society. We therefore took each parent's smoking data from the round subsequent to the offspring's birth in which they reported the earliest onset. Responses regarding the parent's smoking history were combined with data on the offspring and parent's date of birth to infer the parent's smoking status (categorical; never-smoker, ex-smoker, or current smoker) at the time of the offspring's conception and the age at which they began smoking. For all smoking data, smoking was considered to consist in smoking at least one cigarette daily. Responses regarding the parents' educational level (<10 years, 10–12 years, or >12 years), employment type (unskilled, skilled/clerical, farmer/fisher, or professional), BMI, alcohol consumption (< once per fortnight, 1–4 times per fortnight, or ≥5 times per fortnight), physical activity (none, light, or heavy) and blood pressure were taken from the same HUNT round as their smoking history unless they were missing in that round, in which case they were taken from the earliest HUNT round post-dating the offspring's birth. A binary variable was derived from the available data indicating whether the offspring was the oldest of their mother's participating offspring. Binary variables were also derived indicating if a person was in professional employment and if they had completed secondary education (≥10 years).

Two individuals were excluded from further analysis because their dates of birth were inconsistent with their mothers' and one individual was excluded because the identity codes for both parents were missing. The resulting dataset of 66,243 offspring was used in sensitivity analyses after multiple imputation (see below). For the main analyses of paternal smoking onset age, individuals were excluded if their father did not participate in HUNT (13,115 exclusions) or if data were unavailable for the offspring's BMI (1,339 exclusions) or for the father's smoking onset age (4,958 exclusions). Missing data on offspring birth order (3,011 cases), maternal education (5,507 cases), paternal education (2,401 cases) or paternal employment (3,581 cases) were treated as an additional category and offspring lacking this information were not excluded. This gave a final sample of 23,758 sons and 23,073 daughters, of whom 113 sons and 108 daughters had a father who began smoking before 11.

### Statistical analysis

The data included, on average, 1.84 offspring from each father. To avoid the pseudoreplication which would result from the analysis of these siblings as independent observations, each observation was weighted by the reciprocal of family size, such that the sum of weights for each father was equal to one. Family size was defined as the number of offspring included in the analysis who had the same father (or the same mother, if the father was unidentified). This weighting was applied in all analyses.

Demographic and behavioural variables in mothers, fathers and offspring were summarised according to the father's age of smoking onset. Unadjusted weighted linear or logistic regression models were used to predict each demographic or behavioural variable from paternal onset age, and a post-hoc test of the equality of the coefficients for each category of onset age was used to test for any association.

Offspring BMI was first summarised without adjustment within categories defined by the father's age of smoking onset and the offspring's sex and age at BMI measurement. Weighting was applied within each sex and age class of offspring. The categories of paternal smoking onset age were (i) <11 years, (ii) 11–12, (iii) 13–14, (iv) 15+, and (v) never. Offspring age at measurement was first categorised into eight-year bands from age 12 (with those over 35 combined due to low sample size), but in a sensitivity analysis aimed at replicating more closely a previous study [5], offspring were restricted to teenagers, placed into two-year age brackets. To examine secular trends in the exposure and outcome, trends in parental smoking onset age and offspring BMI were plotted against five-year bands of offspring date of birth.

Subsequently, paternal onset age was dichotomised according to whether or not the father began smoking before the age of 11 years and weighted linear regressions of offspring BMI against this dichotomous variable were conducted separately for sons, daughters, and offspring of either sex. Primary analyses were conducted for offspring of all ages, with adjustment for offspring birth order, maternal education, paternal employment, both parents' smoking status at the time of the offspring's conception and a restricted cubic spline of offspring age (knots at the 5^th^, 27.5^th^, 50^th^, 72.5^th^ and 95^th^ percentiles) [13]. The combined analysis of sons and daughters was also adjusted for offspring sex. To test whether associations with paternal smoking onset age differed between sons and daughters, an interaction term between sex and the dichotomous exposure was added to the combined analysis. To examine whether associations in the primary analyses were driven by offspring of particular ages, the analyses were repeated without adjustment for offspring age, within each age group previously defined (including the teenage groups). As sensitivity analyses, they were also repeated without adjustment, and with additional adjustment for (i) paternal and maternal smoking status at offspring conception and (ii) a linear term for offspring date of birth. The power of our unadjusted analysis to detect the effect sizes found for sons in the ALSPAC study was assessed with α = 0.05 using the mean differences in son's BMI according to whether or not the father smoked by 11 reported in [14] as effect sizes. These were combined with the standard deviations and weighted sample sizes from sons of the most closely corresponding age classes (12–13, 14–15, 16–17 and all teenagers) in our data.

The scarcity of mothers or grandparents who began smoking early made a full repetition of the analysis for these ancestors impossible, but the unadjusted summary of offspring BMI by categories of ancestral smoking onset age was repeated for mothers and for grandfathers (early-smoking grandmothers were too scarce even for this). Inclusion in this analysis required participation in HUNT and exposure data for the ancestor in question, rather than the father. Family identity for the weighting was defined primarily according to the mother's identity for maternal and maternal grandfather exposures, and by the father's identity for paternal grandfather exposures.

In a series of sensitivity analyses, we compared weighted linear regressions of offspring BMI against paternal smoking onset by age 11 with and without additional control variables that potentially captured offspring or parent self-control problems (always requiring non-missing data for that variable). These additional control variables were: (i) offspring smoking status at the time of BMI measurement (never-, ex- or current smokers); (ii) The father's BMI at the time their smoking history was recorded; (iii) The father's self-reported intoxication in the two weeks prior to participation (HUNT1 only, 41% yes among fathers); (iv) The father's self-reported intoxication in the four weeks prior to participation (HUNT3 only, 16% yes among fathers); (v) The offspring's response to "have you seen your parents drunk" (YH only, 39% never, 38% a few times, 24% a few times a year or more); (vi) The father's status as an eldest child. These models were also adjusted for the standard set of terms described for the main analyses of offspring BMI, and were applied to offspring of all ages combined.

To test whether the results were biased by the exclusion of those HUNT participants with missing data or unidentified parents, missing values for the outcome, exposures, and covariates were assumed "missing at random" [15] and imputed 100 times using multivariate imputation by the chained equations method (see S1 Table for details). A separate imputation with a reduced set of variables was used to impute data for stratification by offspring age, because the rarity of some binary variables (as well as the exposure) otherwise resulted in perfect prediction when data were stratified by age. Demographic and behavioural characteristics of parents and offspring were summarised in (i) all non-missing data, (ii) all HUNT participants, with missing data imputed and (iii) all participating and non-participating parents, with missing data imputed. The unadjusted description of participants' BMI, demographic and behavioural characteristics within categories of paternal smoking onset age was then repeated using results averaged over the imputed datasets. The estimation of mean differences in offspring BMI according to whether or not the father began smoking before 11 years old was repeated as described above, except that results from each imputed dataset were combined using Rubin's rules [16] [17]. Additionally, the analyses were repeated on a strict complete case subset of the main dataset. In this subset, subjects with missing data for birth order, maternal education, paternal education or paternal employment were omitted instead of missing data in these adjustment variables being treated as an additional category. Whereas multiply imputed data is expected to be less vulnerable to selection bias, this strict complete case subset is expected to be more vulnerable than the main analysis. All analyses were performed using Stata 14.1.

## Results

### Paternal smoking onset

Table 1 reports the characteristics of fathers, their partners and their offspring, according to the age at which the father started smoking. In the data, around 66% of fathers smoked at some point in time, but only 0.4% of fathers started smoking before age 11. There was a suggestion of increasing diversity in smoking onset age, with those starting aged 11–14, or never smoking, being born later than those taking up smoking after the age of 15. Smoking onset was socioeconomically patterned, with those starting later or never (and their partners and offspring) more likely to be in professional employment, to have completed secondary education, and to be older at their offspring's birth, although the age-at-birth pattern seems to reverse for the earliest onset age. Multiple imputation resulted in a somewhat earlier-born cohort of fathers and more offspring who had completed secondary education (S2 Table). Despite imputation increasing the sample size from 25,469 to 36,380 fathers, most characteristics of parents and offspring (S2 Table and S3 Table) were very similar to those in the non-missing data (Table 1), including the socioeconomic patterning of paternal smoking onset reported above.

**Table 1:** Characteristics of fathers, mothers and offspring according to the father's age of smoking onset.

		N_sw_	Father's age of smoking onset						
Variable	N_raw_		<11 years	11–12 years	13–14 years	≥15 years	Never	P_all_	P_ever_
*Means (SD) or percentages in the father*:									
Date of birth	46,831	25,469	1934.9 (15.8)	1941.4 (16.9)	1944.4 (14.9)	1936.9 (15.8)	1941.2 (19.2)	<0.001	<0.001
Age at participation	46,831	25,469	56.7 (14.8)	51.5 (15.9)	49.9 (15.0)	54.4 (14.7)	48.8 (16.3)	<0.001	<0.001
Age at offspring birth	46,831	25,469	30.5 (6.3)	28.8 (6.3)	28.3 (5.9)	30.2 (6.1)	30.7 (6.0)	<0.001	<0.001
BMI (kg m^-2^)	46,657	25,380	26.3 (3.8)	26.1 (3.5)	26.6 (3.7)	26.1 (3.4)	25.9 (3.1)	<0.001	<0.001
Professional employment	43,250	23,532	23%	18%	25%	29%	36%	<0.001	<0.001
Full secondary education	44,430	24,014	31%	39%	48%	47%	62%	<0.001	<0.001
Current smoker	46,831	25,469	92%	87%	87%	85%	0%	99.000	0.029
Drink ≥ fortnightly	46,031	25,071	62%	66%	67%	60%	50%	<0.001	<0.001
*Means (SD) or percentages in the mother*:									
Date of birth	43,820	23,765	1939.1 (14.5)	1944.8 (15.9)	1947.6 (14.3)	1940.7 (15.1)	1945.5 (17.5)	<0.001	<0.001
Age at participation	42,115	22,869	50.2 (14.5)	44.5 (13.9)	43.4 (13.2)	49.1 (13.8)	45.1 (14.7)	<0.001	<0.001
Age at offspring birth	43,820	23,765	26.9 (5.7)	25.6 (5.2)	25.5 (5.2)	26.9 (5.5)	27.6 (5.4)	<0.001	<0.001
BMI (kg m^-2^)	43,463	23,560	26.4 (5.0)	25.6 (4.9)	25.3 (4.8)	25.8 (4.6)	25.5 (4.5)	<0.001	0.004
Professional employment	37,245	20,460	5%	14%	19%	20%	32%	<0.001	0.001
Full secondary education	41,324	22,293	31%	45%	51%	43%	60%	<0.001	<0.001
Current smoker	42,115	22,869	51%	53%	58%	44%	22%	<0.001	<0.001
Drink ≥ fortnightly	42,164	22,891	34%	35%	41%	38%	36%	0.002	0.102
*Means (SD) or percentages in the offspring*:									
Date of birth	46,831	25,469	1965.4 (14.7)	1970.2 (14.6)	1972.8 (13.7)	1967.2 (14.5)	1971.9 (17.3)	<0.001	<0.001
Age at participation	46,831	25,469	27.5 (10.0)	25.8 (9.7)	24.1 (8.7)	27.3 (9.6)	25.2 (10.8)	<0.001	<0.001
BMI (kg m^-2^)	46,831	25,469	24.6 (4.6)	24.1 (4.8)	23.9 (4.4)	24.0 (4.1)	23.3 (3.9)	<0.001	0.302
Professional employment	33,098	18,603	20%	20%	20%	30%	30%	<0.001	<0.001
Full secondary education	28,344	15,561	74%	73%	81%	80%	79%	0.015	0.067
Current smoker	44,531	24,795	37%	36%	32%	29%	16%	<0.001	0.005
Drink ≥ fortnightly	35,330	19,366	54%	54%	61%	62%	57%	<0.001	0.093
Male sex	46,831	25,469	52%	45%	50%	51%	51%	0.407	0.329
Maximum N_raw_	46,831		221	413	2,029	28,959	15,209		
Maximum N_sw_		25,469	113	209	1,150	15,439	8,558		

Current smoker for parents is inferred smoking status at the time of the offspring's birth and for offspring it is from the time of BMI measurement. Observations (N_raw_) were weighted by the reciprocal of the number of siblings (of either sex and age) analysed and N_sw_ is the sum of weights. P values are from unadjusted linear or logistic regressions of the variables against categories of paternal smoking onset age. P_ever_ only compared the ever-smoking categories. All sons and daughters included in the main analyses, and their parents, are included here.

Table 2 displays sons’ and daughters’ mean BMI by paternal smoking onset age. When offspring of all ages were considered together, there was some indication of increased BMI among the daughters of earlier-smoking fathers, but there was no comparable overall pattern among sons. The separation of offspring into different age groups suggested that early paternal smoking was most strongly associated with daughters' BMI when they were younger. For better comparison with a previous study [5], the 12–19 age class was broken down into finer categories (S6 Table). The association of early paternal smoking with BMI among 12–19 year old daughters was mostly driven by daughters younger than 16, although sample sizes were particularly reduced in this higher-resolution analysis. We did not find evidence that the raw association between BMI and early paternal smoking was greater among older daughters. Results among the imputed data were similar to those among the non-missing data, with perhaps a slightly greater indication that the increased BMI among younger daughters of early-smoking fathers might be repeated among sons (S4 Table). Repetition of the main analysis on the strict complete case data subset (in which those with missing data for adjustment variables were omitted) led to slightly stronger associations between early-onset paternal smoking and BMI among daughters (S16 Table).

**Table 2:** Unadjusted mean (SD) offspring BMI at various ages, according to father's age of smoking onset.

Offspring sex; father's onset age	All ages			Offspring 12–19			Offspring 20–27			Offspring 28–35			Offspring 36–76		
	N_raw_	N_sw_	Mean (SD)	N_raw_	N_sw_	Mean (SD)	N_raw_	N_sw_	Mean (SD)	N_raw_	N_sw_	Mean (SD)	N_raw_	N_sw_	Mean (SD)
*Sons*															
<11 years	113	77	24.7 (4.4)	23	19	21.7 (3.0)	31	29	23.1 (2.6)	31	24	25.4 (2.8)	28	22	28.1 (5.8)
11–12 years	191	130	24.3 (4.5)	41	40	22.2 (5.3)	58	48	24.3 (3.5)	56	46	25.7 (4.3)	36	29	26.7 (3.7)
13–14 years	1,013	748	24.1 (4.0)	331	299	22.0 (3.6)	294	255	24.3 (3.3)	241	213	25.9 (3.7)	147	113	26.8 (3.7)
> = 15 years	14,703	10,515	24.4 (3.8)	2,985	2,659	21.7 (3.5)	4,289	3,693	24.4 (3.3)	4,359	3,727	25.4 (3.3)	3,070	2,610	26.2 (3.6)
Never	7,738	5,695	23.7 (3.8)	2,821	2,452	21.5 (3.4)	1,817	1,576	24.2 (3.3)	1,420	1,237	25.3 (3.3)	1,680	1,305	26.0 (3.2)
*Daughters*															
<11 years	108	74	24.6 (5.0)	21	21	23.6 (4.0)	32	24	25.1 (4.9)	35	28	24.7 (5.4)	20	17	25.2 (6.1)
11–12 years	222	153	23.8 (4.8)	62	52	21.6 (3.7)	66	54	24.9 (5.2)	63	52	24.4 (4.7)	31	25	25.3 (3.7)
13–14 years	1,016	759	23.7 (4.7)	349	306	22.3 (4.2)	307	273	24.0 (4.9)	230	198	24.6 (4.5)	130	109	25.7 (5.5)
> = 15 years	14,256	10,277	23.7 (4.2)	3,018	2,630	21.9 (3.6)	4,458	3,867	23.6 (3.9)	4,132	3,549	24.2 (4.2)	2,648	2,285	25.0 (4.4)
Never	7,471	5,502	23.1 (4.0)	2,767	2,378	21.6 (3.3)	1,929	1,697	23.6 (3.9)	1,383	1,198	24.2 (4.2)	1,392	1,124	25.0 (4.2)

Observations in all analyses were weighted by the reciprocal of the number of siblings (of the specified sex and age) used in that analysis, N_raw_ is the unweighted sample size, and N_sw_ is the sum of weights. The power to detect the effect size reported in the ALSPAC study for 12–19 year old sons with α = 0.05 was 94.7%

Following the analysis in [1], BMI among those offspring whose fathers started smoking before the age of 11 is compared with the rest of the population in Table 3, with adjustment for offspring birth order, maternal and paternal education and paternal employment. The mean differences for sons, daughters and the combined sexes were estimated with rather low precision, but there was no evidence overall that they differed between sons and daughters (all ages, P_interaction_ = 0.427). In the combined analyses of sons and daughters, paternal smoking before 11 years was consistently associated with higher offspring BMI, but the 95% confidence intervals excluded the null within only one (≥36 years) of the four categories of offspring age, and included the null when all ages of offspring were considered. When offspring of all ages were analysed together, but separately for sons and daughters, there was very weak evidence suggesting that paternal smoking before 11 was associated with higher BMI among daughters but not among sons (mean difference in BMI (95% confidence interval) of 0.97 (-0.06, 1.87) and 0.22 (-0.53, 0.97), respectively). Once again, the greater BMI among the daughters of men who began smoking before 11 appeared to be driven by those up to 27 years old and the association among 12–19 year old daughters appeared to be driven by girls younger than 16 years of age (S7 Table). Sons' BMI was not associated with paternal smoking before 11 overall, but there was some evidence suggesting that individuals aged 20–27 were slightly *less* heavy if their father started smoking early and an opposite result for sons aged 36 and over. These results did not form any consistent pattern with son's age, and the results for particular age categories should be considered in the context of the number of age-specific tests conducted. The equivalent results from the imputed data were broadly similar, but the more extreme results from the non-missing data tended to be attenuated among the imputed data (S5 Table). There was thus no substantial evidence in the latter for an association between offspring BMI and paternal smoking before 11 at any age, for sons or daughters, except for a weak positive association among sons aged 36 and over.

**Table 3:** Mean difference (95% confidence interval) in offspring BMI at various ages, if the father began smoking before 11 years old.

	All ages, adjusted	Offspring 12–19	Offspring 20–27	Offspring 28–35	Offspring 36–76
*Sons and daughters*					
N_raw_	221 / 46,831	44 / 12,418	63 / 13,281	66 / 11,950	48 / 9,182
N_sw_	112.5 / 25,469	35 / 9,473	46 / 10,157	43 / 8,927	33 / 6,568
MD (95% CI)	0.58 (-0.11, 1.26)	0.76 (-0.40, 1.92)	0.26 (-0.81, 1.33)	0.48 (-0.68, 1.63)	1.73 (0.38, 3.08)
P	0.098	0.199	0.633	0.417	0.012
P_interaction_	0.427	0.230	0.007	0.592	0.130
*Sons*					
N_raw_	113 / 23,758	23 / 6,201	31 / 6,489	31 / 6,107	28 / 4,961
N_sw_	77 / 17,165	19 / 5,469	29 / 5,601	24 / 5,247	22 / 4,079
MD (95% CI)	0.22 (-0.53, 0.97)	-0.18 (-1.75, 1.39)	-1.23 (-2.44, -0.02)	0.12 (-1.22, 1.46)	2.00 (0.54, 3.47)
P	0.570	0.820	0.046	0.864	0.007
*Daughters*					
N_raw_	108 / 23,073	21 / 6,217	32 / 6,792	35 / 5,843	20 / 4,221
N_sw_	73.5 / 16,765	21 / 5,386	24 / 5,915	28 / 5,025	17 / 3,560
MD (95% CI)	0.97 (0.06, 1.87)	1.50 (0.00, 3.00)	1.45 (-0.14, 3.05)	0.74 (-0.84, 2.32)	0.15 (-1.93, 2.23)
P	0.036	0.050	0.074	0.358	0.886

Linear regressions were adjusted for eldest offspring status, mother's and father's education level and father's employment type. Observations in all analyses were weighted by the reciprocal of the number of siblings (of the specified sex and age) used in that analysis, and N_sw_ is the sum of weights for those whose fathers began smoking before 11 years old, followed by the total sum of weights. N_raw_ are the unweighted sample sizes. One father of two daughters reported different onset ages in the first HUNT wave following each birth, giving rise to the non-integer N_sw_ among cases. The analysis of all offspring ages was additionally adjusted for a cubic spline of offspring age. P_interaction_ tests whether the MD differs between sons and daughters.

### Maternal and grandparental smoking onset

Unfortunately, the data contained very few mothers who began smoking early, such that the analyses described above for fathers do not yield reliable estimates for the association with an early smoking onset among mothers. However, none of the raw differences indicated a strong difference in offspring BMI by maternal smoking onset (S9 Table). The same is true if we investigate the effect of early smoking among maternal or paternal grandfathers (S10 and S11 Tables).

### The potential role of confounding

Additional adjustment in the main analyses for variables potentially representing self-control in parents or offspring did not substantially alter estimates of the association between offspring BMI and paternal smoking before 11 years old (S12 Table). Many of these variables were only available for a subset of the data, however, and the restriction of the analyses to these smaller samples did change the estimates considerably, with or without the additional adjustment. In addition, confounding may originate from secular trends both in the probability of an early smoking onset and in child BMI. S1 Fig shows that raw offspring BMI declined over the study period while BMI corrected for the offspring’s age increased (linear regression for raw or age-adjusted BMI; P<0.001 in sons and daughters). The secular trend in early paternal smoking onset depended on the age threshold used, declining (P = 0.014), remaining approximately constant (P = 0.574), or increasing (P<0.001) for age thresholds of 11, 13 and 15 years, respectively. Maternal early onset smoking showed dramatic increases (P = 0.052, P<0.001 and P<0.001 for thresholds of 11, 13 and 15 years, respectively), albeit from a very low starting point. Despite these clear secular trends, estimates of the association between early paternal smoking onset and offspring BMI were not substantially changed by additional adjustment for offspring date of birth (S15 Table). Additional adjustment for maternal and paternal smoking status at the time of the offspring's conception had a small attenuating effect on the already weak association among sons and slightly amplified the positive association among daughters. When offspring of all ages were analysed with no adjustment at all, except for a cubic spline of age at measurement, the estimated associations between offspring BMI and paternal smoking onset age changed very little, though there was some movement among the imprecise age-stratified results (S13 Table).

## Discussion

Using a different dataset in an attempt to replicate earlier findings [5], this paper provides at best weak evidence in support of an effect of paternal smoking onset during the slow growth period on offspring BMI. The only association apparent in the data was between early (age < 11) paternal smoking and daughter’s BMI, which seems to be driven by the younger age groups. Our findings clearly do not support the hypothesis of an effect of early paternal smoking on sons' BMI and the already weak association with daughters’ BMI should be considered alongside the parallel tests of sons and, to some extent, of maternal onset age.

Arguably, we cannot fully rule out intergenerational effects of early paternal smoking on offspring BMI. First, the treatment group in our analysis is small and very selective, as only 0.4% of fathers started smoking before age 11. Given such a small sample of exposed individuals the estimates are never precisely zero and positive BMI effects of reasonable magnitude might exist according to the estimated confidence intervals. However, given our findings and the estimated confidence intervals, effect sizes for sons in the order of 2–3 BMI points as reported in [1] are very unlikely. Second, we found that the association between early paternal smoking onset and daughter’s BMI persisted after controlling for a considerable number of variables related to parental socio-economic status, parental self-control, and parallel trends in smoking and BMI. Third, we might miss a positive causal effect of early paternal smoking on offspring BMI, because such an effect is counteracted by the children’s own smoking behaviour, as we find children of fathers who started smoking early (before age 13) to be more likely to smoke at age 15 than children of fathers who started smoking later (S8 Table). Smoking is associated with appetite suppressions and reduced BMI [18] [19] [20]. This might also explain why we found early-onset paternal smoking to be weakly associated with lower BMI in sons aged 20–29, i.e., during prime smoking age. Nevertheless, we conclude that an intergenerational epigenetic mechanism is an unlikely explanation for intergenerational associations between smoking onset and BMI. The reason is that such a mechanism would most likely persist (or increase) over the life cycle. Moreover, it would most likely not involve different sexes of offspring in different populations, but would materialize to a similar extent in sons and daughters or along consistent sex-specific lines [21].

There are a number of weaknesses in this study. First, our sample is selective in a sense that the father and the offspring both need to be HUNT participants. Compared with the population of Nord-Trøndelag, the old, the young, men, the seriously ill and those from lower socioeconomic groups are under-represented in the HUNT data [12] [22] [23]. Further, in the main analyses, we require paternal self-reported smoking onset and several important background variables of parents and children to be non-missing. While this is a common (and unavoidable) limitation to all analyses spanning more than one generation, it may bias the estimated effect of early paternal smoking if unobserved variables that drive selection also confound the causal relationship of interest. Sample selectivity should be less problematic in our case, because we aim to uncover a biological mechanism which should be apparent among all parts of the population as long as the father started smoking during the slow growth period. The slight attenuation of the association among daughters in the imputed data and its amplification in the strict complete case data suggest the presence of some selection bias. However, the small magnitude of the differences, and their absence in the analysis of sons, suggest that selection bias is not a major problem in the main analysis. Second, the sample of fathers with a very early smoking onset is small and most children only enter the study in their 20s and 30s, such that very precise point estimates in the sample of teenage children could not be obtained. Nevertheless, we are able to conclude that the data patterns observed in [5] are unlikely to be the same in our data, especially regarding the effect of paternal smoking on boys’ BMI. Third, we need to estimate the onset age from self-reported information gathered many years after the actual smoking onset. This is relevant as we find some indication that reported smoking onset ages increase in repeated surveys, i.e., as smoking became more stigmatized in society. We dealt with this problem by using the earliest reported smoking onset. Fourth, our data do not contain exogenous variation in smoking onset, such that we can only focus on conditional associations, with varied sets of control variables.

The strengths of the study include its large sample size and the possibility to follow the offspring over a long period of time. In fact our data contain information on measured offspring BMI up to age 76, such that we were able to test the smoking onset hypothesis with offspring BMI data over the entire life-cycle. An additional strength of the study is the comprehensive data set which allowed us to investigate a large number of potential confounders including adjustment variables related to socio-economic status, self-control and paternal smoking at the conception of the study child. Thus we were able to show that parental SES (and to a lesser and non-linear extent parental age) relate to parental smoking onset, which might explain some of the overall patterns observed in the data. Moreover, the correlation between SES and smoking onset might suggest that there are further unmeasured aspects related with SES which confound the association between paternal smoking onset and offspring BMI.

Are inter- or even transgenerational effects of smoking during preadolescence implausible? Certainly not. It has previously been shown in mouse models that exposure to chemicals such as Diethylstilbestrol, Vinclozolin or Methoxyclor during embryonic development can indeed alter gonad development and spermatogenesis of male offspring and that part of this phenotype is iterated in males of subsequent generations through epigenetic modifications of the male gametes [24] [25] [26] [7] [27]. Tobacco smoke in particular leads to many epigenetic modifications, such as the hypermethylation of tumour suppressor genes in non-transformed lung cells [28] [29]. That epigenetic modifications may occur during paternal preadolescence is equally plausible, as the age of preadolescence, (also sometimes called the slow growth period) was found to be a critical period in several related contexts [30] [31] [32]. Epigenetic modifications to the male germ line that alter the metabolism of the next generation are thus plausible, although there are many other possible links through which epigenetic changes may affect obesity and vice versa [11] [33]. However, we do not find strong support for this hypothesis in the data we analysed for this paper.

What else might drive the association found in this paper? First, there might be additional confounders, which are unobservable and largely unrelated to the self-control variables, socio-economic status controls and secular trends available in the HUNT data. Second, our findings have to be interpreted against the fact that parallel tests have been conducted, i.e., for various age groups and two sexes. Against this background, and given that we do not observe any general patterns in the data (such as a consistent trend with respect to offspring age, in the association between offspring BMI and early onset paternal smoking) it is possible that the weak positive coefficients we found were due to chance. Overall, given our findings of a positive association between early smoking and the BMI of daughters, but not of sons and the above discussion, we think that confounding, chance in parallel tests, or sample selection effects are as likely to explain our finding of a weak positive relationship between daughter’s BMI and paternal smoking onset as an epigenetic response is.

Using a large health dataset, we have failed to validate previous findings according to which cigarette smoking in mid childhood is associated with an elevated body mass index (BMI) specifically among male offspring. However, a weaker association, perhaps common to male and female offspring cannot be ruled out. At the same time our findings are specific to one type of exposure (early smoking) and one type of outcome (BMI) and thus additional research may show that exposure to early smoking or other environmental factors results in intergenerational effects of different sorts. Studies that focus on effects across two or more generations in humans are by construction non-experimental and often conducted on relatively small samples. Careful validation studies on other populations are thus useful to advance our knowledge in this important field of research.

## Supporting Information

S1 Fig Secular trends in the exposures and outcome. Offspring BMI was grouped into five-year bands by date of birth, except that those born 1920–1939 were combined due to scarcity. Each band was plotted at its mean date of birth. BMI for age was calculated as residuals from a sex-specific regression of BMI against a cubic spline of age with knots at the 5^th^, 27.5^th^, 50^th^, 72.5^th^ and 95^th^ percentiles. Error bars are 95% confidence intervals.
(DOCX)

S1 Table Variables used in the multiple imputation procedure. (DOCX)

S2 Table Characteristics of fathers, mothers and offspring among non-missing and imputed data. (DOCX)

S3 Table Characteristics of fathers, mothers and offspring according to the father's age of smoking onset in the imputed dataset. (DOCX)

S4 Table Unadjusted mean (SD) offspring BMI at various ages, according to father's age of smoking onset in the imputed dataset. (DOCX)

S5 Table Mean difference (95% confidence interval) in offspring BMI at various ages, if the father began smoking before 11 years old in the imputed dataset. (DOCX)

S6 Table Unadjusted mean (SD) teenage offspring BMI at various ages, according to father's age of smoking onset. (DOCX)

S7 Table Mean difference (95% confidence interval) in teenage offspring BMI at various ages, if the father began smoking before 11 years old. (DOCX)

S8 Table Proportion of offspring smoking early according to paternal age of smoking onset. (DOCX)

S9 Table Unadjusted mean (SD) offspring BMI at various ages, according to mother's age of smoking onset. (DOCX)

S10 Table Unadjusted mean (SD) grand-offspring BMI at various ages, according to paternal grandfather's age of smoking onset. (DOCX)

S11 Table Unadjusted mean (SD) grand-offspring BMI at various ages, according to maternal grandfather's age of smoking onset. (DOCX)

S12 Table Mean differences (95% confidence interval) in offspring BMI if the father began smoking before 11 years old, with and without adjustment for measures of parent or offspring self-control. (DOCX)

S13 Table Unadjusted mean difference (95% confidence interval) in offspring BMI at various ages, if the father began smoking before 11 years old. (DOCX)

S14 Table Mean difference (95% confidence interval) in offspring BMI at various ages, if the father began smoking before 11 years old. Additionally adjusted for parental smoking status at offspring conception.
(DOCX)

S15 Table Mean difference (95% confidence interval) in offspring BMI at various ages, if the father began smoking before 11 years old. Additionally adjusted for a linear association with offspring date of birth.
(DOCX)

S16 Table Mean difference (95% confidence interval) in offspring BMI at various ages, if the father began smoking before 11 years old among a strict complete case subset of the data. (DOCX)

## References

[1] Northstone K, Golding J, Davey Smith G, Miller LL, Pembrey M. Prepubertal start of father's smoking and increased body fat in his sons: further characterisation of peaternal transgenerational responses. European Journal of Human Genetics. 2014: p. 1–5. 10.1038/ejhg.2014.31 PMC4085023 24690679

[2] Pembrey ME, Bygren LO, Kaati G, Edvinsson S, Northstone K, Sjöström M, et al Sex-specific, male-line transgenerational responses in humans. European Journal of Human Genetics 14(2). 2005: p. 159–166. 10.1038/sj.ejhg.5201538 16391557

[3] Kaati G, Bygren LO, Edvinsson S. Cardiovascular and diabetes mortality determined by nutrition during parents' and grandparents' slow growth period. European journal of human genetics 10(11). 2002: p. 682–688. 10.1038/sj.ejhg.5200859 12404098

[4] Bygren LO, Kaati G, Edvinsson S. Longevity determined by paternal ancestors' nutrition during their slow growth period. Acta biotheoretica 49(1). 2001: p. 53–59. 11368478 10.1023/a:1010241825519

[5] Northstone K, Golding J, Davey Smith G, Miller LL, Pembrey M. Prepubertal start of father's smoking and increased body fat in his sons: further characterisation of peaternal transgenerational responses. European Journal of Human Genetics. 2014: p. 1–5. 10.1038/ejhg.2014.31 PMC4085023 24690679

[6] Tobi EW, Lumey L, Talens R, Kremer D, Putter H, Stein AD, et al DNA methylation differences after exposure to prenatal famine are common and timing-and sex-specific. Human Molecular Genetics 18(21). 2009: p. 4046–4053. 10.1093/hmg/ddp353 19656776 PMC2758137

[7] Anway MD, Cupp AS, Uzumcu M, Skinner MK. Epigenetic Transgenerational Actions of Endocrine Disruptors and Male Fertility. Science. 2005: p. 1466–1469. 10.1126/science.1108190 15933200 PMC11423801

[8] Soubry A, Schildkraut JM, Murtha A, Wang F, Huang Z, Bernal A, et al Paternal obesity is associated with IGF2 hypomethylation in newborns: results from a Newborn Epigenetics Study (NEST) cohort. BMC medicine 11(1). 2013: p. 29. 23388414 10.1186/1741-7015-11-29 PMC3584733

[9] Dunn GA, Bale TL. Maternal high-fat diet effects on third-generation female body size via the paternal lineage. Endocrinology 152(6). 2011: p. 2228–2236. 10.1210/en.2010-1461 21447631 PMC3100614

[10] Grossniklaus U, Kelly B, Ferguson-Smith AC, Pembrey M. Transgenerational epigenetic inheritance: how important is it? Nature Reviews Genetics 14 2013: p. 228–235. 10.1038/nrg3435 23416892 PMC4066847

[11] Youngson NA, Morris MJ. What obesity research tells us about epigenetic mechanisms. Philosophical Transactions of the Royal Society B. 2013: p. 368. 10.1098/rstb.2011.0337 PMC3539363 23166398

[12] Krokstad S, Langhammer A, Hveem K, Holmen TL, Midthjell K, Stene TR, et al Cohort profile: the HUNT study, Norway. International journal of epidemiology, 42(4). 2013: p. 968–977. 10.1093/ije/dys095 22879362

[13] Harrell FE. Regression modeling strategies: with applications to linear models, logistic regression, and survival analysis Springer Science & Business Media, 2013. New York: Springer; 2013.

[14] Northstone K, Golding J, Davey Smith G, Miller LL, Pembrey M. Prepubertal start of father's smoking and increased body fat in his sons: further characterisation of peaternal transgenerational responses. European Journal of Human Genetics. 2014: p. 1–5. 10.1038/ejhg.2014.31 PMC4085023 24690679

[15] Sterne JA, White IR, Carlin JB, Spratt M, Royston P, Kenward MG, et al Multiple imputation for missing data in epidemiological and clinical research: potential and pitfalls. British Medical Journal, 339(7713). 2009: p. 157–160. 10.1136/bmj.b2393 PMC2714692 19564179

[16] Rubin Donald B. Multiple imputation for nonresponse in surveys; 1987.

[17] Rubin DB. Multiple imputation after 18+ years. Journal of the American statistical Association, 91(434). 1996: p. 473–489.

[18] Freathy RM, Kazeem GR, Morris RW, Johnson PC, Paternoster L, Ebrahim S, et al Genetic variation at CHRNA5-CHRNA3-CHRNB4 interacts with smoking status to influence body mass index. International journal of epidemiology. 2011: p. dyr077. 10.1093/ije/dyr077 PMC3235017 21593077

[19] Klesges RC, Winders SE, Meyers AW, Eck LH, Ward KD, Hultquist CM, et al How much weight gain occurs following smoking cessation: A comparison of weight gain using both continuous and point prevalence abstinence. Journal of consulting and clinical psychology, 65(2). 1997: p. 286 9086692 10.1037//0022-006x.65.2.286

[20] Williamson DF, Madans J, Anda RF, Kleinman JC, Byers GAGaT. Smoking cessation and severity of weight gain in a national cohort. New England Journal of Medicine 324(11). 1991: p. 739–745. 10.1056/NEJM199103143241106 1997840

[21] Cooney CA. Germ cells carry the epigenetic benefits of grandmother's diet. Proceedings of the National Academy of Sciences, 103 (46). 2006: p. 17071–17072. 10.1073/pnas.0608653103 PMC1859889 17101997

[22] Holmen J, Midthjell K, Forsen L, Skjerve K, Gorseth M, Oseland A. A health survey in Nord-Trondelag 1984–86. Participation and comparison of attendants and non-attendants. Tidsskrift for den Norske laegeforening: tidsskrift for praktisk medicin, ny raekke 110(15). 1990: p. 1973–1977. 2363169

[23] Holmen J, Midthjell K, Krüger Ø, Langhammer A, Holmen TL, Bratberg GH, et al The Nord-Trøndelag Health Study 1995–97 (HUNT 2): objectives, contents, methods and participation. Norsk epidemiologi 13(1). 2003: p. 19–32.

[24] Stouder C, Paoloni-Giacobino A. Transgenerational effects of the endocrine disruptor vinclozolin on the methylation pattern of imprinted genes in the mouse sperm. Reproduction 139 2010: p. 373–379. 10.1530/REP-09-0340 19887539

[25] Stouder C, Paoloni-Giacobino A. Specific transgenerational imprinting effects of the endocrine disruptor methoxychlor on male gametes. Reproduction 141 2011: p. 207–216. 10.1530/REP-10-0400 21062904

[26] Guerrero-Bosagna C, Settles M, Lucker B, Skinner MK. Epigenetic Transgenerational Actions of Vinclozolin on Promoter Regions of the Sperm Epigenome. PLoS ONE 5 2010: p. e13100 10.1371/journal.pone.0013100 20927350 PMC2948035

[27] Feil R, Fraga MF. Epigenetics and the environment: emerging patterns and implications. Nature Reviews Genetics. 2012: p. 97–109. 10.1038/nrg3142 22215131

[28] Belinsky SA, Palmisano WA, Gilliland FD, Crooks LA, Divine KK, Winters SA, et al Aberrant promoter methylation in bronchial epithelium and sputum from current and former smokers. Cancer Research. 2002: p. 2370–2377. 11956099

[29] Breitling LP, Yang R, Korn B, Burwinkel B, Brenner H. Tobacco-smoking-related differential DNA methylation: 27K discovery and replication. The American Journal of Human Genetics. 2011: p. 450–457. 10.1016/j.ajhg.2011.03.003 21457905 PMC3071918

[30] van den Berg GJ, Lundborg P, Nystedt P, Rooth DO. Critical Periods During Childhood And Adolescence. Journal of the European Economic Association. 2014, 12(6): p. 1521–1557.

[31] van den Berg GJ, Pinger PR. Transgenerational Effects of Childhood Conditions on Third Generation Health and Education Outcomes. Economics & Human Biology. 2016, 23: p. 103–120. 27592272 10.1016/j.ehb.2016.07.001

[32] Sparén P, Vagero D, Shestov DB, Plavinskaja S, Parfenova N, Hoptiar V, et al Long term mortality after severe starvation during the siege of Leningrad: prospective cohort study. British Medical Journal 7430(328). 2004: p. 11—14A. 10.1136/bmj.37942.603970.9A PMC313894 14660443

[33] Whitelaw NC, Chong S, Morgan DK, Nestor C, Bruxner TJ, Ashe A, et al Reduced levels of two modifiers of epigenetic gene silencing, Dnmt3a and Trim28, cause increased phenotypic noise. Genome Biology. 2010: p. R111 10.1186/gb-2010-11-11-r111 21092094 PMC3156950

