# Supplementary material for: Early-Onset Paternal Smoking and Offspring Adiposity: Further Investigation of a Potential Intergenerational Effect Using the HUNT Study
Source: PLoS One. 2016 Dec 2;11(12):e0166952. doi: 10.1371/journal.pone.0166952 (PMC5135283; doi:10.1371/journal.pone.0166952)
Supplement: S1 Fig — Offspring BMI was grouped into five-year bands by date of birth, except that those born 1920–1939 were combined due to scarcity. Each band was plotted at its mean date of birth. BMI for age was calculated as residuals from a sex-specific regression of BMI against a cubic spline of age with knots at the 5th, 27.5th, 50th, 72.5th and 95th percentiles. Error bars are 95% confidence intervals. (DOCX) [file pone.0166952.s001.docx]

**Figure S1. Secular trends in the exposures and outcome.** Offspring BMI was grouped into five-year bands by date of birth, except that those born 1920-1939 were combined due to scarcity. Each band was plotted at its mean date of birth. BMI for age was calculated as residuals from a sex-specific regression of BMI against a cubic spline of age with knots at the 5^th^, 27.5^th^, 50^th^, 72.5^th^ and 95^th^ percentiles. Error bars are 95% confidence intervals.
