# Supplementary material for: Early-Onset Paternal Smoking and Offspring Adiposity: Further Investigation of a Potential Intergenerational Effect Using the HUNT Study
Source: PLoS One. 2016 Dec 2;11(12):e0166952. doi: 10.1371/journal.pone.0166952 (PMC5135283; doi:10.1371/journal.pone.0166952)
Supplement: S1 Table — (DOCX) [file pone.0166952.s002.docx]

**Table S1. Variables used in the multiple imputation procedure.**

| Variable | Use in final analysis | Method for imputation of variable | Use in imputation of other variables | Notes |
| --- | --- | --- | --- | --- |
| Body mass index_O_ | Outcome variable | Linear regression | Continuous variable |  |
| Smoking onset age_F_ | Exposure variable | Ordinal logistic regression | 4 indicator variables |  |
| Sex_O_ | Stratifying/descriptive variable | No missing data | Binary stratification variable |  |
| First-born_O_ | Adjustment variable | Logistic regression | Binary variable |  |
| Education category_M/F_ | Adjustment variable | Ordinal logistic regression | 2 indicator variables |  |
| Employment category_F_ | Adjustment variable | Multinomial logistic regression | 6 indicator variables |  |
| Date of birth_O_ | Adjustment variable | No missing data | Continuous variable |  |
| Smoking status at survey_O_ | Sensitivity adjustment variable | Logistic regression | 2 indicator variables | 1,2 |
| Body mass index_M/F_ | Sensitivity adjustment/descriptive variable | Linear regression | Continuous variable |  |
| First-born_M/F_ | Sensitivity adjustment variable | Logistic regression | Binary variable | 2 |
| Date of birth_M/F_ | Descriptive variable | Linear regression | Continuous variable |  |
| Age at participation_O_ | Descriptive variable | No missing data | Continuous variable |  |
| Age at participation_M/F_ | Descriptive variable | Linear regression | Continuous variable |  |
| Age at offspring birth_M/F_ | Descriptive variable | Derived variable | Not used |  |
| Professional employment_O/M_ | Descriptive variable | Logistic Regression | Binary variable | 2 |
| Full secondary education_O_ | Descriptive variable | Derived variable | Not used | 2 |
| Full secondary education_M/F_ | Descriptive variable | Derived variable | Not used |  |
| Regular drinking_O_ | Descriptive variable | Logistic regression | Binary variable | 2 |
| Regular drinking_M/F_ | Descriptive variable | Logistic regression | Binary variable |  |
| Smoking onset age_O/M_ | Descriptive variable | Ordinal logistic regression | Not used |  |
| Education category_O_ | Imputation variable only | Ordinal logistic regression | 2 indicator variables | 2 |
| Exercise_O/M/F_ | Imputation variable only | Ordinal logistic regression | 2 indicator variables | 2 |
| Systolic blood pressure_O/M/F_ | Imputation variable only | Linear regression | Continuous variable |  |

^1^Imputation was restricted to equal 0 (non-smoker) when the offspring's onset age was 5 (have never smoked).

^2^These variables were omitted from the imputation stratified by offspring age

Subscripts O, M and F refer to the offspring, mother and father, respectively.
