# Supplementary material for: Early-Onset Paternal Smoking and Offspring Adiposity: Further Investigation of a Potential Intergenerational Effect Using the HUNT Study
Source: PLoS One. 2016 Dec 2;11(12):e0166952. doi: 10.1371/journal.pone.0166952 (PMC5135283; doi:10.1371/journal.pone.0166952)
Supplement: S2 Table — (DOCX) [file pone.0166952.s003.docx]

**Table S2. Characteristics of fathers, mothers and offspring among non-missing and imputed data.**

|  | Sample size (sum of weights) | | |  | Mean (SD) or percentage | | |
| --- | --- | --- | --- | --- | --- | --- | --- |
| Variable | Non-missing | Partici-pants | All |  | Non-missing | Participants | All |
| *In the father:* |  |  |  |  |  |  |  |
| Smoker by 11 | 25,867 | 28,607 | 36,380 |  | 0.4% | 0.4% | 0.4% |
| Smoker by 13 | 25,867 | 28,607 | 36,380 |  | 1.2% | 1.2% | 1.2% |
| Smoker by 15 | 25,867 | 28,607 | 36,380 |  | 5.8% | 5.7% | 5.7% |
| Ever-smoker | 25,867 | 28,607 | 36,380 |  | 66% | 66% | 66% |
| Date of birth | 28,607 | 28,607 | 36,380 |  | 1938.1 (17.7) | 1938.1 (17.7) | 1935.7 (19.2) |
| Age at participation | 25,867 | 28,607 | 36,380 |  | 52.2 (15.5) | 52.9 (15.9) | 54.9 (17.0) |
| Age at offspring birth | 28,607 | 28,607 | 36,380 |  | 30.5 (6.2) | 30.5 (6.2) | 30.6 (6.3) |
| BMI (kg m^-2^) | 27,879 | 28,607 | 36,380 |  | 26.0 (3.4) | 26.0 (3.4) | 26.0 (3.4) |
| Professional employment | 24,587 | 28,607 | 36,380 |  | 31% | 30% | 29% |
| Full secondary education | 25,082 | 28,607 | 36,380 |  | 52% | 52% | 49% |
| Current smoker | 25,867 | 25,867 | 25,867 |  | 56% | 56% | 56% |
| Drink ≥ fortnightly | 26,243 | 28,607 | 36,380 |  | 57% | 56% | 53% |
| Recently intoxicated, HUNT1 | 13,108 | 13,108 | 13,108 |  | 41% | 41% | 41% |
| Recently intoxicated, HUNT3 | 10,837 | 10,837 | 10,837 |  | 18% | 18% | 18% |
|  |  |  |  |  |  |  |  |
| *In the mother:* |  |  |  |  |  |  |  |
| Smoker by 11 | 31,709 | 34,212 | 36,380 |  | 0.0% | 0.0% | 0.0% |
| Smoker by 13 | 31,709 | 34,212 | 36,380 |  | 0.4% | 0.4% | 0.4% |
| Smoker by 15 | 31,709 | 34,212 | 36,380 |  | 3.2% | 3.1% | 3.1% |
| Ever-smoker | 31,709 | 34,212 | 36,380 |  | 49% | 48% | 48% |
| Date of birth | 34,212 | 34,212 | 36,380 |  | 1939.4 (18.7) | 1939.4 (18.7) | 1938.9 (19.0) |
| Age at participation | 31,709 | 34,212 | 36,380 |  | 50.2 (15.8) | 51.1 (16.2) | 51.5 (16.5) |
| Age at offspring birth | 34,212 | 34,212 | 36,380 |  | 27.4 (5.7) | 27.4 (5.7) | 27.4 (5.7) |
| BMI (kg m^-2^) | 33,518 | 34,212 | 36,380 |  | 25.9 (4.6) | 25.9 (4.6) | 25.9 (4.6) |
| Professional employment | 27,084 | 34,212 | 36,380 |  | 23% | 21% | 21% |
| Full secondary education | 30,471 | 34,212 | 36,380 |  | 45% | 45% | 44% |
| Current smoker | 31,709 | 31,709 | 31,709 |  | 36% | 36% | 36% |
| Drink ≥ fortnightly | 31,771 | 34,212 | 36,380 |  | 34% | 33% | 33% |
| Recently intoxicated, HUNT1 | 10,271 | 10,271 | 10,271 |  | 16% | 16% | 16% |
| Recently intoxicated, HUNT3 | 13,082 | 13,082 | 13,082 |  | 6% | 6% | 6% |
|  |  |  |  |  |  |  |  |
| *In the offspring:* |  |  |  |  |  |  |  |
| Smoker by 11 | 35,098 | 36,380 | 36,380 |  | 0.2% | 0.2% | 0.2% |
| Smoker by 13 | 35,098 | 36,380 | 36,380 |  | 1.0% | 1.0% | 1.0% |
| Smoker by 15 | 35,098 | 36,380 | 36,380 |  | 5.8% | 5.7% | 5.7% |
| Ever-smoker | 35,098 | 36,380 | 36,380 |  | 40% | 40% | 40% |
| Date of birth | 36,380 | 36,380 | 36,380 |  | 1966.3 (17.5) | 1966.3 (17.5) | 1966.3 (17.5) |
| Age at participation | 36,380 | 36,380 | 36,380 |  | 28.2 (11.4) | 28.2 (11.4) | 28.2 (11.4) |
| BMI (kg m^-2^) | 35,795 | 36,380 | 36,380 |  | 24.0 (4.1) | 23.9 (4.1) | 23.9 (4.1) |
| Professional employment | 26,465 | 36,380 | 36,380 |  | 30% | 20% | 20% |
| Full secondary education | 22,932 | 36,380 | 36,380 |  | 74% | 82% | 82% |
| Current smoker | 35,098 | 36,380 | 36,380 |  | 27% | 27% | 27% |
| Drink ≥ fortnightly | 27,608 | 36,380 | 36,380 |  | 59% | 61% | 61% |
| Recently intoxicated, HUNT1 | 10,839 | 10,839 | 10,839 |  | 45% | 45% | 45% |
| Recently intoxicated, HUNT3 | 18,872 | 18,872 | 18,872 |  | 24% | 24% | 24% |
| Seen parents intoxicated, YH | 11,943 | 11,960 | 12,011 |  | 63% | 63% | 63% |
| Male sex | 36,380 | 36,380 | 36,380 |  | 51% | 51% | 51% |
