# Supplementary material for: Early-Onset Paternal Smoking and Offspring Adiposity: Further Investigation of a Potential Intergenerational Effect Using the HUNT Study
Source: PLoS One. 2016 Dec 2;11(12):e0166952. doi: 10.1371/journal.pone.0166952 (PMC5135283; doi:10.1371/journal.pone.0166952)
Supplement: S3 Table — (DOCX) [file pone.0166952.s004.docx]

**Table S3. Characteristics of fathers, mothers and offspring according to the father's age of smoking onset in the imputed dataset.**

|  |  | N_sw_ | Father's age of smoking onset | | | | |  |  |
| --- | --- | --- | --- | --- | --- | --- | --- | --- | --- |
| Variable | N_raw_ |  | <11 years | 11-12 years | 13-14 years | ≥15 years | Never | P_all_ | P_ever_ |
| *Means (SD) or percentages in the father:* | | | | | | | | | |
| Date of birth | 66,243 | 36,380 | 1932.7 (17.3) | 1937.5 (18.7) | 1939.8 (18.1) | 1934.3 (18.1) | 1937.8 (21.1) | <0.001 | <0.001 |
| Age at participation | 66,243 | 36,380 | 59.0 (16.0) | 55.2 (17.2) | 53.9 (16.9) | 56.7 (16.3) | 51.8 (17.9) | <0.001 | <0.001 |
| Age at offspring birth | 66,243 | 36,380 | 30.4 (6.3) | 29.3 (6.4) | 29.0 (6.1) | 30.5 (6.3) | 31.0 (6.3) | <0.001 | <0.001 |
| BMI (kg m^-2^) | 66,243 | 36,380 | 26.2 (3.7) | 26.2 (3.5) | 26.5 (3.6) | 26.1 (3.4) | 25.9 (3.2) | <0.001 | <0.001 |
| Professional employment | 66,243 | 36,380 | 24% | 18% | 24% | 28% | 33% | <0.001 | <0.001 |
| Full secondary education | 66,243 | 36,380 | 30% | 36% | 44% | 45% | 58% | <0.001 | 0.001 |
| Current smoker | 48,023 | 25,867 | 92% | 87% | 87% | 85% | 0% | 99.000 | 0.038 |
| Drink ≥ fortnightly | 66,243 | 36,380 | 60% | 63% | 64% | 56% | 47% | <0.001 | <0.001 |
|  |  |  |  |  |  |  |  |  |  |
| *Means (SD) or percentages in the mother:* | | | | | | | | | |
| Date of birth | 66,243 | 36,380 | 1936.2 (17.4) | 1940.9 (18.2) | 1942.7 (18.0) | 1937.5 (18.0) | 1940.8 (20.8) | <0.001 | <0.001 |
| Age at participation | 66,243 | 36,380 | 53.6 (16.2) | 49.2 (16.2) | 48.3 (16.1) | 52.6 (15.9) | 50.0 (17.5) | <0.001 | <0.001 |
| Age at offspring birth | 66,243 | 36,380 | 26.9 (5.7) | 25.8 (5.5) | 26.0 (5.5) | 27.2 (5.7) | 27.9 (5.7) | <0.001 | <0.001 |
| BMI (kg m^-2^) | 66,243 | 36,380 | 26.3 (4.9) | 25.8 (4.8) | 25.6 (4.8) | 26.0 (4.6) | 25.8 (4.6) | 0.015 | 0.029 |
| Professional employment | 66,243 | 36,380 | 7% | 13% | 17% | 18% | 27% | <0.001 | 0.006 |
| Full secondary education | 66,243 | 36,380 | 28% | 39% | 44% | 39% | 53% | <0.001 | <0.001 |
| Current smoker | 57,814 | 31,709 | 46% | 48% | 52% | 41% | 24% | <0.001 | <0.001 |
| Drink ≥ fortnightly | 66,243 | 36,380 | 32% | 33% | 37% | 34% | 31% | <0.001 | 0.057 |
|  |  |  |  |  |  |  |  |  |  |
| *Means (SD) or percentages in the offspring:* | | | | | | | | | |
| Date of birth | 66,243 | 36,380 | 1963.1 (16.0) | 1966.8 (16.4) | 1968.7 (16.5) | 1964.7 (16.5) | 1968.8 (19.2) | <0.001 | <0.001 |
| Age at participation | 66,243 | 36,380 | 29.1 (11.0) | 27.9 (10.9) | 26.6 (10.7) | 28.9 (11.0) | 27.2 (12.2) | <0.001 | <0.001 |
| BMI (kg m^-2^) | 66,243 | 36,380 | 24.6 (4.5) | 24.3 (4.7) | 24.1 (4.4) | 24.1 (4.1) | 23.5 (4.0) | <0.001 | 0.561 |
| Professional employment | 66,243 | 36,380 | 20% | 20% | 20% | 20% | 20% | 0.041 | 0.017 |
| Full secondary education | 66,243 | 36,380 | 76% | 79% | 84% | 81% | 84% | <0.001 | 0.108 |
| Current smoker | 66,243 | 36,380 | 41% | 40% | 37% | 31% | 18% | <0.001 | <0.001 |
| Drink ≥ fortnightly | 66,243 | 36,380 | 56% | 58% | 62% | 62% | 58% | <0.001 | 0.449 |
| Male sex | 66,243 | 36,380 | 52% | 44% | 50% | 51% | 52% | 0.171 | 0.162 |
|  |  |  |  |  |  |  |  |  |  |
| Maximum N_raw_ | 66,243 |  | 309 | 570 | 2,905 | 40,944 | 21,514 |  |  |
| Maximum N_sw_ |  | 36,380 | 160 | 294 | 1,631 | 22,102 | 12,193 |  |  |

Current smoker for parents is inferred smoking status at the time of the offspring's birth and for offspring it is from the time of BMI measurement. Observations (N_raw_) were weighted by the reciprocal of the number of siblings (of either sex and age) analysed and N_sw_ is the sum of weights. P values are from unadjusted linear or logistic regressions of the variables against categories of paternal smoking onset age. P_ever_ only compared the ever-smoking categories.
