# Supplementary material for: Early-Onset Paternal Smoking and Offspring Adiposity: Further Investigation of a Potential Intergenerational Effect Using the HUNT Study
Source: PLoS One. 2016 Dec 2;11(12):e0166952. doi: 10.1371/journal.pone.0166952 (PMC5135283; doi:10.1371/journal.pone.0166952)
Supplement: S4 Table — (DOCX) [file pone.0166952.s005.docx]

**Table S4. Unadjusted mean (SD) offspring BMI at various ages, according to father's age of smoking onset in the imputed dataset.**

| Offspring sex; father's onset age | All ages | | |  | Offspring 12-19 | | |  | Offspring 20-27 | | |  | Offspring 28-35 | | |  | Offspring 36-76 | | |
| --- | --- | --- | --- | --- | --- | --- | --- | --- | --- | --- | --- | --- | --- | --- | --- | --- | --- | --- | --- |
|  | N_raw_ | N_sw_ | Mean (SD) |  | N_raw_ | N_sw_ | Mean (SD) |  | N_raw_ | N_sw_ | Mean (SD) |  | N_raw_ | N_sw_ | Mean (SD) |  | N_raw_ | N_sw_ | Mean (SD) |
| *Sons* |  |  |  |  |  |  |  |  |  |  |  |  |  |  |  |  |  |  |  |
| <11 years | 157 | 108 | 24.7 (4.3) |  | 32 | 26 | 21.6 (3.3) |  | 41 | 38 | 23.4 (2.8) |  | 39 | 31 | 25.4 (2.9) |  | 45 | 36 | 27.4 (5.1) |
| 11-12 years | 248 | 172 | 24.4 (4.4) |  | 52 | 50 | 22.2 (5.1) |  | 71 | 59 | 24.3 (3.5) |  | 73 | 60 | 25.6 (4.2) |  | 53 | 42 | 26.6 (3.6) |
| 13-14 years | 1,345 | 986 | 24.2 (4.0) |  | 430 | 383 | 22.1 (3.7) |  | 365 | 316 | 24.3 (3.3) |  | 325 | 284 | 25.8 (3.6) |  | 225 | 176 | 26.6 (3.7) |
| >=15 years | 20,710 | 14,743 | 24.5 (3.8) |  | 3,919 | 3,451 | 21.8 (3.6) |  | 5,331 | 4,603 | 24.3 (3.3) |  | 5,868 | 5,011 | 25.3 (3.3) |  | 5,591 | 4,567 | 26.2 (3.5) |
| Never | 11,417 | 8,370 | 23.9 (3.8) |  | 3,665 | 3,183 | 21.6 (3.5) |  | 2,249 | 1,955 | 24.2 (3.3) |  | 1,940 | 1,683 | 25.2 (3.3) |  | 3,563 | 2,734 | 25.9 (3.3) |
|  |  |  |  |  |  |  |  |  |  |  |  |  |  |  |  |  |  |  |  |
| *Daughters* |  |  |  |  |  |  |  |  |  |  |  |  |  |  |  |  |  |  |  |
| <11 years | 143 | 100 | 24.5 (4.9) |  | 27 | 27 | 23.4 (4.0) |  | 39 | 30 | 24.9 (4.8) |  | 46 | 38 | 24.7 (5.2) |  | 31 | 26 | 25.3 (5.7) |
| 11-12 years | 292 | 206 | 23.8 (4.7) |  | 83 | 70 | 21.9 (3.8) |  | 82 | 68 | 24.7 (5.0) |  | 82 | 69 | 24.3 (4.7) |  | 45 | 37 | 25.3 (4.0) |
| 13-14 years | 1,356 | 1,008 | 23.8 (4.7) |  | 457 | 398 | 22.4 (4.1) |  | 394 | 350 | 24.0 (4.8) |  | 306 | 264 | 24.6 (4.6) |  | 200 | 167 | 25.6 (5.2) |
| >=15 years | 19,822 | 14,372 | 23.8 (4.3) |  | 3,970 | 3,460 | 22.0 (3.6) |  | 5,597 | 4,884 | 23.6 (4.0) |  | 5,567 | 4,810 | 24.1 (4.3) |  | 4,688 | 3,912 | 25.1 (4.4) |
| Never | 10,752 | 7,966 | 23.4 (4.1) |  | 3,610 | 3,111 | 21.6 (3.3) |  | 2,382 | 2,098 | 23.6 (3.9) |  | 1,869 | 1,628 | 24.1 (4.2) |  | 2,891 | 2,289 | 25.2 (4.3) |

Observations in all analyses were weighted by the reciprocal of the number of siblings (of the specified sex and age) used in that analysis, N_raw_ is the unweighted sample size, and N_sw_ is the sum of weights.
