# Supplementary material for: Early-Onset Paternal Smoking and Offspring Adiposity: Further Investigation of a Potential Intergenerational Effect Using the HUNT Study
Source: PLoS One. 2016 Dec 2;11(12):e0166952. doi: 10.1371/journal.pone.0166952 (PMC5135283; doi:10.1371/journal.pone.0166952)
Supplement: S6 Table — (DOCX) [file pone.0166952.s007.docx]

**Table S6. Unadjusted mean (SD) teenage offspring BMI at various ages, according to father's age of smoking onset.**

| Offspring sex; father's onset age | All ages | | |  | Offspring 12-13 | | |  | Offspring 14-15 | | |  | Offspring 16-17 | | |  | Offspring 18-19 | | |
| --- | --- | --- | --- | --- | --- | --- | --- | --- | --- | --- | --- | --- | --- | --- | --- | --- | --- | --- | --- |
|  | N_raw_ | N_sw_ | Mean (SD) |  | N_raw_ | N_sw_ | Mean (SD) |  | N_raw_ | N_sw_ | Mean (SD) |  | N_raw_ | N_sw_ | Mean (SD) |  | N_raw_ | N_sw_ | Mean (SD) |
| *Sons* |  |  |  |  |  |  |  |  |  |  |  |  |  |  |  |  |  |  |  |
| <11 years | 113 | 77 | 24.7 (4.4) |  | 5 | 5 | 21.5 (4.2) |  | 5 | 5 | 22.0 (2.9) |  | 9 | 9 | 21.6 (3.0) |  | 4 | 4 | 22.2 (1.7) |
| 11-12 years | 191 | 130 | 24.3 (4.5) |  | 7 | 7 | 19.4 (2.7) |  | 15 | 15 | 21.7 (4.8) |  | 13 | 13 | 23.8 (7.3) |  | 6 | 5 | 23.4 (1.9) |
| 13-14 years | 1,013 | 748 | 24.1 (4.0) |  | 54 | 54 | 19.8 (2.7) |  | 133 | 132 | 21.7 (3.5) |  | 97 | 96 | 22.7 (3.8) |  | 47 | 46 | 23.4 (3.2) |
| >=15 years | 14,703 | 10,515 | 24.4 (3.8) |  | 494 | 485 | 20.1 (3.4) |  | 1,085 | 1,065 | 21.1 (3.3) |  | 901 | 883 | 22.3 (3.6) |  | 505 | 499 | 23.3 (3.3) |
| Never | 7,738 | 5,695 | 23.7 (3.8) |  | 434 | 431 | 19.8 (3.2) |  | 1,040 | 1,019 | 20.8 (3.1) |  | 901 | 874 | 22.3 (3.3) |  | 446 | 435 | 23.2 (3.4) |
|  |  |  |  |  |  |  |  |  |  |  |  |  |  |  |  |  |  |  |  |
| *Daughters* |  |  |  |  |  |  |  |  |  |  |  |  |  |  |  |  |  |  |  |
| <11 years | 108 | 74 | 24.6 (5.0) |  | 2 | 2 | 23.6 (7.7) |  | 7 | 7 | 24.0 (4.9) |  | 8 | 8 | 22.6 (3.4) |  | 4 | 4 | 24.9 (2.0) |
| 11-12 years | 222 | 153 | 23.8 (4.8) |  | 11 | 11 | 20.2 (1.6) |  | 24 | 24 | 21.7 (4.2) |  | 14 | 14 | 21.9 (4.1) |  | 13 | 13 | 21.2 (2.9) |
| 13-14 years | 1,016 | 759 | 23.7 (4.7) |  | 58 | 58 | 20.8 (3.7) |  | 127 | 122 | 21.5 (4.1) |  | 104 | 102 | 23.0 (3.9) |  | 60 | 59 | 24.3 (4.1) |
| >=15 years | 14,256 | 10,277 | 23.7 (4.2) |  | 473 | 467 | 20.5 (3.5) |  | 1,097 | 1,066 | 21.4 (3.3) |  | 861 | 838 | 22.6 (3.6) |  | 587 | 583 | 23.2 (3.6) |
| Never | 7,471 | 5,502 | 23.1 (4.0) |  | 458 | 451 | 20.2 (3.0) |  | 1,004 | 977 | 21.1 (3.1) |  | 817 | 793 | 22.2 (3.3) |  | 488 | 479 | 22.7 (3.0) |

Observations in all analyses were weighted by the reciprocal of the number of siblings (of the specified sex and age) used in that analysis, N_raw_ is the unweighted sample size, and N_sw_ is the sum of weights. The power to detect the effect sizes reported for sons in the ALSPAC study with α=0.05 was 18.6% for 12-13 year olds, 26.0% for 14-15 year olds and 84.0% for 16-17 year olds, respectively.
