# Supplementary material for: Early-Onset Paternal Smoking and Offspring Adiposity: Further Investigation of a Potential Intergenerational Effect Using the HUNT Study
Source: PLoS One. 2016 Dec 2;11(12):e0166952. doi: 10.1371/journal.pone.0166952 (PMC5135283; doi:10.1371/journal.pone.0166952)
Supplement: S8 Table — (DOCX) [file pone.0166952.s009.docx]

**Table S8. Proportion of offspring smoking early according to paternal age of smoking onset.**

| Age father started smoking |  |  |  |  |  | % (N_sw_) smoking at 11 | |  | % (N_sw_) smoking at 13 | |  | % (N_sw_) smoking at 15 | |
| --- | --- | --- | --- | --- | --- | --- | --- | --- | --- | --- | --- | --- | --- |
|  | N_raw, daughters_ | N_raw, sons_ | N_sw, daughters_ | N_sw, sons_ |  | Daughters | Sons |  | Daughters | Sons |  | Daughters | Sons |
| <11 years | 103 | 110 | 69.0 | 74.5 |  | 0.0% (0.0) | 0.0% (0.0) |  | 1.4% (1.0) | 2.0% (1.5) |  | 11.2% (7.7) | 8.9% (6.7) |
| 11-12 years | 211 | 180 | 144.3 | 124.2 |  | 0.0% (0.0) | 0.4% (0.5) |  | 3.3% (4.8) | 1.6% (2.0) |  | 17.5% (25.2) | 10.6% (13.2) |
| 13-14 years | 962 | 961 | 719.8 | 707.3 |  | 0.4% (2.8) | 0.2% (1.2) |  | 1.6% (11.3) | 1.6% (11.0) |  | 11.0% (78.9) | 11.1% (78.4) |
| >=15 years | 13,623 | 13,914 | 9,806.1 | 9,946.3 |  | 0.1% (10.0) | 0.2% (23.0) |  | 0.9% (87.7) | 1.0% (101.4) |  | 6.3% (620.4) | 5.7% (566.7) |
| Never | 7,122 | 7,345 | 5,242.4 | 5,396.6 |  | 0.1% (2.8) | 0.2% (13.2) |  | 0.3% (14.7) | 0.8% (42.2) |  | 3.2% (165.7) | 3.1% (166.8) |
|  |  |  |  |  |  |  |  |  |  |  |  |  |  |
| P_all_ |  |  |  |  |  | 0.048 | 0.959 |  | <0.001 | 0.202 |  | <0.001 | <0.001 |
| P_ever_ |  |  |  |  |  | 0.045 | 0.874 |  | 0.014 | 0.438 |  | <0.001 | <0.001 |

P values are from logistic regressions of offspring smoking onset by the specified age against categories of paternal onset age. Paternal never-smokers were either included (P_all_) or excluded (P_ever_) from these analyses. N_raw_ are the unweighted sample sizes and N_sw_ are the sums of weights.
