# Supplementary material for: Early-Onset Paternal Smoking and Offspring Adiposity: Further Investigation of a Potential Intergenerational Effect Using the HUNT Study
Source: PLoS One. 2016 Dec 2;11(12):e0166952. doi: 10.1371/journal.pone.0166952 (PMC5135283; doi:10.1371/journal.pone.0166952)
Supplement: S9 Table — (DOCX) [file pone.0166952.s010.docx]

**Table S9. Unadjusted mean (SD) offspring BMI at various ages, according to mother's age of smoking onset.**

| Offspring sex; mother's onset age | All ages | | |  | Offspring 12-19 | | |  | Offspring 20-27 | | |  | Offspring 28-35 | | |  | Offspring 36-76 | | |
| --- | --- | --- | --- | --- | --- | --- | --- | --- | --- | --- | --- | --- | --- | --- | --- | --- | --- | --- | --- |
|  | N_raw_ | N_sw_ | Mean (SD) |  | N_raw_ | N_sw_ | Mean (SD) |  | N_raw_ | N_sw_ | Mean (SD) |  | N_raw_ | N_sw_ | Mean (SD) |  | N_raw_ | N_sw_ | Mean (SD) |
| *Sons* |  |  |  |  |  |  |  |  |  |  |  |  |  |  |  |  |  |  |  |
| <11 years | 12 | 8 | 23.8 (3.9) |  | 6 | 5 | 22.1 (3.1) |  | 3 | 3 | 25.8 (3.6) |  | 0 | 0 |  |  | 3 | 1 | 26.7 (18332.3) |
| 11-12 years | 68 | 55 | 23.3 (4.5) |  | 44 | 39 | 21.8 (4.2) |  | 18 | 15 | 25.4 (3.4) |  | 3 | 3 | 29.0 (5.0) |  | 3 | 3 | 27.4 (1.2) |
| 13-14 years | 605 | 494 | 23.0 (4.3) |  | 425 | 370 | 21.8 (3.6) |  | 110 | 103 | 25.0 (4.6) |  | 50 | 48 | 27.6 (4.1) |  | 20 | 20 | 28.5 (5.2) |
| >=15 years | 12,250 | 9,069 | 24.3 (4.0) |  | 3,522 | 3,122 | 21.9 (3.7) |  | 3,697 | 3,220 | 24.5 (3.4) |  | 3,152 | 2,715 | 25.7 (3.5) |  | 1,879 | 1,630 | 26.8 (3.7) |
| Never | 15,701 | 10,950 | 24.3 (3.6) |  | 3,006 | 2,633 | 21.4 (3.4) |  | 3,390 | 2,914 | 24.0 (3.0) |  | 4,069 | 3,455 | 25.0 (3.1) |  | 5,236 | 4,095 | 25.9 (3.3) |
|  |  |  |  |  |  |  |  |  |  |  |  |  |  |  |  |  |  |  |  |
| *Daughters* |  |  |  |  |  |  |  |  |  |  |  |  |  |  |  |  |  |  |  |
| <11 years | 16 | 11 | 23.0 (3.5) |  | 6 | 6 | 22.1 (4.5) |  | 5 | 5 | 23.2 (2.5) |  | 1 | 1 | 27.2 (0.0) |  | 4 | 3 | 22.9 (2.0) |
| 11-12 years | 74 | 63 | 23.9 (5.3) |  | 53 | 47 | 22.8 (5.2) |  | 15 | 12 | 27.0 (4.5) |  | 5 | 5 | 26.2 (4.5) |  | 1 | 1 | 20.5 (0.0) |
| 13-14 years | 679 | 556 | 23.2 (4.6) |  | 437 | 378 | 22.3 (4.0) |  | 170 | 158 | 25.0 (5.1) |  | 54 | 47 | 24.5 (5.5) |  | 18 | 17 | 24.9 (4.1) |
| >=15 years | 12,612 | 9,276 | 23.7 (4.4) |  | 3,599 | 3,152 | 22.0 (3.6) |  | 4,082 | 3,567 | 23.9 (4.2) |  | 3,224 | 2,774 | 24.6 (4.6) |  | 1,707 | 1,499 | 25.5 (4.6) |
| Never | 14,377 | 10,227 | 23.5 (4.1) |  | 3,009 | 2,578 | 21.5 (3.3) |  | 3,399 | 2,960 | 23.2 (3.7) |  | 3,654 | 3,151 | 23.7 (3.8) |  | 4,315 | 3,453 | 24.9 (4.4) |

Observations in all analyses were weighted by the reciprocal of the number of siblings (of the specified sex and age) used in that analysis, N_raw_ is the unweighted sample size, and N_sw_ is the sum of weights.
