# Supplementary material for: Early-Onset Paternal Smoking and Offspring Adiposity: Further Investigation of a Potential Intergenerational Effect Using the HUNT Study
Source: PLoS One. 2016 Dec 2;11(12):e0166952. doi: 10.1371/journal.pone.0166952 (PMC5135283; doi:10.1371/journal.pone.0166952)
Supplement: S10 Table — (DOCX) [file pone.0166952.s011.docx]

**Table S10. Unadjusted mean (SD) grand-offspring BMI at various ages, according to paternal grandfather's age of smoking onset.**

| Offspring sex; ancestor's onset age | All ages | | |  | Offspring 12-19 | | |  | Offspring 20-27 | | |  | Offspring 28-35 | | |  | Offspring 36-76 | | |
| --- | --- | --- | --- | --- | --- | --- | --- | --- | --- | --- | --- | --- | --- | --- | --- | --- | --- | --- | --- |
|  | N_raw_ | N_sw_ | Mean (SD) |  | N_raw_ | N_sw_ | Mean (SD) |  | N_raw_ | N_sw_ | Mean (SD) |  | N_raw_ | N_sw_ | Mean (SD) |  | N_raw_ | N_sw_ | Mean (SD) |
| *Sons* |  |  |  |  |  |  |  |  |  |  |  |  |  |  |  |  |  |  |  |
| <11 years | 31 | 23 | 23.1 (4.5) |  | 13 | 12 | 21.2 (4.5) |  | 13 | 10 | 25.2 (4.2) |  | 4 | 4 | 23.9 (1.7) |  | 1 | 1 | 31.1 (0.0) |
| 11-12 years | 57 | 44 | 23.9 (4.5) |  | 39 | 34 | 23.1 (4.4) |  | 13 | 11 | 25.5 (2.4) |  | 4 | 4 | 29.4 (5.0) |  | 1 | 1 | 21.9 (0.0) |
| 13-14 years | 181 | 148 | 23.1 (4.2) |  | 115 | 101 | 21.6 (3.1) |  | 40 | 37 | 24.1 (3.2) |  | 20 | 18 | 27.0 (4.3) |  | 6 | 5 | 31.7 (6.0) |
| >=15 years | 4,200 | 3,358 | 22.9 (4.0) |  | 2,750 | 2,396 | 21.7 (3.6) |  | 924 | 833 | 24.6 (3.6) |  | 409 | 373 | 26.4 (3.7) |  | 117 | 116 | 27.7 (3.9) |
| Never | 1,869 | 1,427 | 23.4 (4.1) |  | 955 | 824 | 21.8 (3.5) |  | 567 | 489 | 24.6 (3.6) |  | 260 | 232 | 26.3 (4.0) |  | 87 | 85 | 27.3 (3.6) |
|  |  |  |  |  |  |  |  |  |  |  |  |  |  |  |  |  |  |  |  |
| *Daughters* |  |  |  |  |  |  |  |  |  |  |  |  |  |  |  |  |  |  |  |
| <11 years | 28 | 22 | 23.5 (3.1) |  | 14 | 11 | 21.7 (2.5) |  | 9 | 9 | 25.5 (3.2) |  | 4 | 4 | 23.1 (2.1) |  | 1 | 1 | 24.3 (0.0) |
| 11-12 years | 56 | 43 | 23.4 (4.3) |  | 36 | 31 | 23.2 (4.4) |  | 11 | 10 | 23.1 (2.3) |  | 6 | 5 | 25.9 (6.7) |  | 3 | 3 | 22.9 (0.1) |
| 13-14 years | 203 | 160 | 23.2 (4.4) |  | 125 | 112 | 22.0 (3.3) |  | 58 | 53 | 24.4 (5.2) |  | 15 | 14 | 29.1 (5.4) |  | 5 | 5 | 25.4 (2.7) |
| >=15 years | 4,536 | 3,551 | 22.9 (4.2) |  | 2,774 | 2,394 | 21.8 (3.5) |  | 1,213 | 1,099 | 24.2 (4.2) |  | 429 | 382 | 25.8 (5.2) |  | 120 | 115 | 26.4 (5.8) |
| Never | 1,946 | 1,470 | 23.1 (4.2) |  | 940 | 810 | 21.6 (3.3) |  | 613 | 532 | 24.2 (4.3) |  | 288 | 258 | 24.9 (4.8) |  | 105 | 101 | 25.2 (4.5) |

Observations in all analyses were weighted by the reciprocal of the number of siblings (of the specified sex and age) used in that analysis, N_raw_ is the unweighted sample size, and N_sw_ is the sum of weights.
