# Supplementary material for: Early-Onset Paternal Smoking and Offspring Adiposity: Further Investigation of a Potential Intergenerational Effect Using the HUNT Study
Source: PLoS One. 2016 Dec 2;11(12):e0166952. doi: 10.1371/journal.pone.0166952 (PMC5135283; doi:10.1371/journal.pone.0166952)
Supplement: S11 Table — (DOCX) [file pone.0166952.s012.docx]

**Table S11. Unadjusted mean (SD) grand-offspring BMI at various ages, according to maternal grandfather's age of smoking onset.**

| Offspring sex; ancestor's onset age | All ages | | |  | Offspring 12-19 | | |  | Offspring 20-27 | | |  | Offspring 28-35 | | |  | Offspring 36-76 | | |
| --- | --- | --- | --- | --- | --- | --- | --- | --- | --- | --- | --- | --- | --- | --- | --- | --- | --- | --- | --- |
|  | N_raw_ | N_sw_ | Mean (SD) |  | N_raw_ | N_sw_ | Mean (SD) |  | N_raw_ | N_sw_ | Mean (SD) |  | N_raw_ | N_sw_ | Mean (SD) |  | N_raw_ | N_sw_ | Mean (SD) |
| *Sons* |  |  |  |  |  |  |  |  |  |  |  |  |  |  |  |  |  |  |  |
| <11 years | 54 | 43 | 23.9 (5.1) |  | 32 | 28 | 22.3 (4.4) |  | 11 | 11 | 24.3 (4.7) |  | 7 | 7 | 29.2 (7.1) |  | 4 | 4 | 29.3 (3.3) |
| 11-12 years | 54 | 42 | 23.6 (4.0) |  | 29 | 24 | 22.9 (3.9) |  | 14 | 12 | 22.8 (2.7) |  | 8 | 7 | 26.5 (5.0) |  | 3 | 3 | 29.1 (3.9) |
| 13-14 years | 265 | 219 | 23.2 (4.9) |  | 190 | 165 | 22.0 (4.3) |  | 46 | 42 | 24.9 (3.5) |  | 21 | 21 | 27.5 (5.9) |  | 8 | 8 | 30.7 (4.8) |
| >=15 years | 4,746 | 3,804 | 23.1 (4.0) |  | 2,932 | 2,571 | 21.8 (3.6) |  | 1,165 | 1,018 | 24.8 (3.6) |  | 463 | 425 | 26.2 (3.8) |  | 186 | 172 | 27.6 (3.8) |
| Never | 2,054 | 1,543 | 23.3 (4.0) |  | 978 | 842 | 21.5 (3.5) |  | 608 | 524 | 24.4 (3.3) |  | 345 | 305 | 25.9 (3.5) |  | 123 | 116 | 27.2 (4.0) |
|  |  |  |  |  |  |  |  |  |  |  |  |  |  |  |  |  |  |  |  |
| *Daughters* |  |  |  |  |  |  |  |  |  |  |  |  |  |  |  |  |  |  |  |
| <11 years | 38 | 30 | 23.1 (4.3) |  | 24 | 22 | 22.2 (3.7) |  | 9 | 8 | 24.1 (5.2) |  | 5 | 5 | 28.3 (4.2) |  | 0 | 0 |  |
| 11-12 years | 72 | 56 | 23.2 (4.7) |  | 41 | 35 | 21.4 (2.8) |  | 17 | 14 | 25.5 (5.6) |  | 11 | 10 | 26.0 (6.3) |  | 3 | 3 | 25.9 (2.0) |
| 13-14 years | 252 | 206 | 23.3 (4.5) |  | 151 | 137 | 22.2 (3.8) |  | 73 | 63 | 24.7 (4.8) |  | 23 | 19 | 25.2 (4.1) |  | 5 | 5 | 29.7 (4.2) |
| >=15 years | 5,244 | 4,082 | 23.0 (4.1) |  | 3,043 | 2,619 | 21.8 (3.5) |  | 1,454 | 1,305 | 24.2 (4.2) |  | 582 | 519 | 25.4 (4.6) |  | 165 | 158 | 25.9 (4.7) |
| Never | 2,087 | 1,565 | 23.1 (4.3) |  | 977 | 842 | 21.7 (3.5) |  | 644 | 570 | 23.8 (4.2) |  | 359 | 322 | 25.1 (5.0) |  | 107 | 100 | 25.3 (4.2) |

Observations in all analyses were weighted by the reciprocal of the number of siblings (of the specified sex and age) used in that analysis, N_raw_ is the unweighted sample size, and N_sw_ is the sum of weights.
