# Supplementary material for: Early-Onset Paternal Smoking and Offspring Adiposity: Further Investigation of a Potential Intergenerational Effect Using the HUNT Study
Source: PLoS One. 2016 Dec 2;11(12):e0166952. doi: 10.1371/journal.pone.0166952 (PMC5135283; doi:10.1371/journal.pone.0166952)
Supplement: S12 Table — (DOCX) [file pone.0166952.s013.docx]

**Table S12. Mean differences (95% confidence interval) in offspring BMI if the father began smoking before 11 years old, with and without adjustment for measures of parent or offspring self-control.**

| Control variable, offspring sex |  |  | Without control variable | |  | With control variable | |
| --- | --- | --- | --- | --- | --- | --- | --- |
|  | N_raw_ | N_sw_ | MD (95% CI) | P |  | MD (95% CI) | P |
| *No additional adjustment (see Table 3)* | | | | | | | |
| Combined | 221 / 46,831 | 112.5 / 25,469 | 0.58 (-0.11, 1.26) | 0.098 |  |  |  |
| Sons | 113 / 23,758 | 77 / 17,165 | 0.22 (-0.53, 0.97) | 0.570 |  |  |  |
| Daughters | 108 / 23,073 | 73.5 / 16,765 | 0.97 (0.06, 1.87) | 0.036 |  |  |  |
|  |  |  |  |  |  |  |  |
| *Offspring smoking status* | | | | | | | |
| Combined | 213 / 44,531 | 106.5 / 24,795 | 0.72 (0.02, 1.43) | 0.044 |  | 0.75 (0.05, 1.46) | 0.035 |
| Sons | 110 / 22,510 | 75 / 16,493 | 0.27 (-0.49, 1.03) | 0.490 |  | 0.31 (-0.45, 1.07) | 0.424 |
| Daughters | 103 / 22,021 | 69.5 / 16,186 | 1.14 (0.21, 2.07) | 0.017 |  | 1.17 (0.24, 2.10) | 0.014 |
|  |  |  |  |  |  |  |  |
| *Parent's BMI (kg m^-2^)* | | | | | | | |
| Combined | 215 / 46,657 | 111.5 / 25,380 | 0.60 (-0.09, 1.29) | 0.087 |  | 0.57 (-0.10, 1.24) | 0.097 |
| Sons | 108 / 23,667 | 76 / 17,100 | 0.25 (-0.51, 1.00) | 0.525 |  | 0.24 (-0.50, 0.98) | 0.521 |
| Daughters | 107 / 22,990 | 72.5 / 16,712 | 1.01 (0.10, 1.92) | 0.030 |  | 0.99 (0.10, 1.88) | 0.030 |
|  |  |  |  |  |  |  |  |
| *Parent intoxicated (HUNT1)* | | | | | | | |
| Combined | 144 / 23,093 | 69.5 / 12,426 | 1.06 (0.19, 1.92) | 0.016 |  | 1.04 (0.17, 1.90) | 0.018 |
| Sons | 77 / 11,742 | 49 / 8,438 | 0.56 (-0.37, 1.50) | 0.238 |  | 0.54 (-0.39, 1.47) | 0.257 |
| Daughters | 67 / 11,351 | 45.5 / 8,245 | 1.57 (0.43, 2.71) | 0.007 |  | 1.55 (0.41, 2.69) | 0.008 |
|  |  |  |  |  |  |  |  |
| *Parent intoxicated (HUNT3)* | | | | | | | |
| Combined | 56 / 18,377 | 31.5 / 10,558 | 0.61 (-0.63, 1.86) | 0.333 |  | 0.57 (-0.68, 1.81) | 0.372 |
| Sons | 32 / 9,033 | 24 / 6,855 | 0.00 (-1.32, 1.31) | 0.997 |  | -0.06 (-1.38, 1.25) | 0.923 |
| Daughters | 24 / 9,344 | 18.5 / 6,982 | 1.89 (0.18, 3.61) | 0.031 |  | 1.82 (0.11, 3.54) | 0.037 |
|  |  |  |  |  |  |  |  |
| *Seen parents intoxicated* | | | | | | | |
| Combined | 42 / 11,957 | 33 / 9,236 | 0.77 (-0.38, 1.92) | 0.188 |  | 0.70 (-0.45, 1.85) | 0.234 |
| Sons | 23 / 5,971 | 19 / 5,276 | -0.40 (-1.89, 1.09) | 0.600 |  | -0.44 (-1.92, 1.05) | 0.564 |
| Daughters | 19 / 5,986 | 19 / 5,225 | 1.73 (0.19, 3.26) | 0.027 |  | 1.62 (0.09, 3.15) | 0.038 |
|  |  |  |  |  |  |  |  |
| *Parent oldest in family* | | | | | | | |
| Combined | 69 / 18,148 | 36.5 / 10,914 | 0.52 (-0.68, 1.71) | 0.397 |  | 0.52 (-0.68, 1.71) | 0.398 |
| Sons | 39 / 8,802 | 26 / 6,875 | 0.16 (-1.16, 1.48) | 0.811 |  | 0.16 (-1.16, 1.48) | 0.813 |
| Daughters | 30 / 9,346 | 24.5 / 7,125 | 0.97 (-0.58, 2.52) | 0.221 |  | 0.97 (-0.58, 2.52) | 0.220 |

Linear regressions were also adjusted for eldest offspring status, mother's and father's education level, father's employment type and a cubic spline of offspring age. Observations in all analyses were weighted by the reciprocal of the number of siblings (of the specified sex and age) used in that analysis, N_raw_ is the unweighted sample size, and N_sw_ is the sum of weights for those whose fathers began smoking before 11 years old, followed by the total sum of weights. Offspring of all ages were included.
