# Supplementary material for: Early-Onset Paternal Smoking and Offspring Adiposity: Further Investigation of a Potential Intergenerational Effect Using the HUNT Study
Source: PLoS One. 2016 Dec 2;11(12):e0166952. doi: 10.1371/journal.pone.0166952 (PMC5135283; doi:10.1371/journal.pone.0166952)
Supplement: S13 Table — (DOCX) [file pone.0166952.s014.docx]

**Table S13. Unadjusted mean difference (95% confidence interval) in offspring BMI at various ages, if the father began smoking before 11 years old.**

|  | All ages, adjusted | Offspring 12-19 | Offspring 20-27 | Offspring 28-35 | Offspring 36-76 |
| --- | --- | --- | --- | --- | --- |
| *Sons and daughters* | |  |  |  |  |
| N_raw_ | 221 / 46,831 | 44 / 12,418 | 63 / 13,281 | 66 / 11,950 | 48 / 9,182 |
| N_sw_ | 112.5 / 25,469 | 35 / 9,473 | 46 / 10,157 | 43 / 8,927 | 33 / 6,568 |
| MD (95% CI) | 0.59 (-0.10, 1.27) | 1.00 (-0.17, 2.16) | 0.27 (-0.80, 1.35) | 0.34 (-0.81, 1.50) | 1.73 (0.38, 3.08) |
| P | 0.094 | 0.094 | 0.621 | 0.560 | 0.012 |
| P_interaction_ | 0.447 | 0.222 | 0.009 | 0.732 | 0.155 |
|  |  |  |  |  |  |
| *Sons* |  |  |  |  |  |
| N_raw_ | 113 / 23,758 | 23 / 6,201 | 31 / 6,489 | 31 / 6,107 | 28 / 4,961 |
| N_sw_ | 77 / 17,165 | 19 / 5,469 | 29 / 5,601 | 24 / 5,247 | 22 / 4,079 |
| MD (95% CI) | 0.24 (-0.51, 0.99) | 0.06 (-1.51, 1.64) | -1.19 (-2.40, 0.02) | 0.07 (-1.27, 1.41) | 1.93 (0.47, 3.40) |
| P | 0.532 | 0.940 | 0.054 | 0.920 | 0.010 |
|  |  |  |  |  |  |
| *Daughters* |  |  |  |  |  |
| N_raw_ | 108 / 23,073 | 21 / 6,217 | 32 / 6,792 | 35 / 5,843 | 20 / 4,221 |
| N_sw_ | 73.5 / 16,765 | 21 / 5,386 | 24 / 5,915 | 28 / 5,025 | 17 / 3,560 |
| MD (95% CI) | 0.97 (0.06, 1.87) | 1.78 (0.28, 3.29) | 1.43 (-0.17, 3.03) | 0.52 (-1.06, 2.10) | 0.21 (-1.87, 2.29) |
| P | 0.037 | 0.020 | 0.079 | 0.516 | 0.842 |

Observations in all analyses were weighted by the reciprocal of the number of siblings (of the specified sex and age) used in that analysis, and N_sw_ is the sum of weights for those whose fathers began smoking before 11 years old, followed by the total sum of weights. N_raw_ are the unweighted sample sizes. The analysis of all offspring ages was additionally adjusted for a cubic spline of offspring age. P_interaction_ tests whether the MD differs between sons and daughters.
