# Supplementary material for: Early-Onset Paternal Smoking and Offspring Adiposity: Further Investigation of a Potential Intergenerational Effect Using the HUNT Study
Source: PLoS One. 2016 Dec 2;11(12):e0166952. doi: 10.1371/journal.pone.0166952 (PMC5135283; doi:10.1371/journal.pone.0166952)
Supplement: S14 Table — Additionally adjusted for parental smoking status at offspring conception. (DOCX) [file pone.0166952.s015.docx]

**Table S14. Mean difference (95% confidence interval) in offspring BMI at various ages, if the father began smoking before 11 years old. Additionally adjusted for maternal and paternal smoking at offspring conception.**

|  | All ages, adjusted | Offspring 12-19 | Offspring 20-27 | Offspring 28-35 | Offspring 36-76 |
| --- | --- | --- | --- | --- | --- |
| *Sons and daughters* | |  |  |  |  |
| N_raw_ | 221 / 46,831 | 44 / 12,418 | 63 / 13,281 | 66 / 11,950 | 48 / 9,182 |
| N_sw_ | 112.5 / 25,469 | 35 / 9,473 | 46 / 10,157 | 43 / 8,927 | 33 / 6,568 |
| MD (95% CI) | 0.57 (-0.16, 1.30) | 0.68 (-0.55, 1.91) | 0.25 (-0.82, 1.33) | 0.33 (-0.86, 1.53) | 2.06 (0.51, 3.60) |
| P | 0.124 | 0.278 | 0.645 | 0.584 | 0.009 |
| P_interaction_ | 0.222 | 0.326 | 0.005 | 0.419 | 0.241 |
|  |  |  |  |  |  |
| *Sons* |  |  |  |  |  |
| N_raw_ | 113 / 23,758 | 23 / 6,201 | 31 / 6,489 | 31 / 6,107 | 28 / 4,961 |
| N_sw_ | 77 / 17,165 | 19 / 5,469 | 29 / 5,601 | 24 / 5,247 | 22 / 4,079 |
| MD (95% CI) | 0.04 (-0.76, 0.85) | -0.21 (-1.87, 1.44) | -1.32 (-2.53, -0.10) | -0.34 (-1.77, 1.09) | 2.30 (0.58, 4.02) |
| P | 0.916 | 0.803 | 0.034 | 0.640 | 0.009 |
|  |  |  |  |  |  |
| *Daughters* |  |  |  |  |  |
| N_raw_ | 108 / 23,073 | 21 / 6,217 | 32 / 6,792 | 35 / 5,843 | 20 / 4,221 |
| N_sw_ | 73.5 / 16,765 | 21 / 5,386 | 24 / 5,915 | 28 / 5,025 | 17 / 3,560 |
| MD (95% CI) | 1.12 (0.17, 2.07) | 1.46 (-0.16, 3.08) | 1.51 (-0.08, 3.09) | 0.71 (-0.89, 2.32) | 0.67 (-1.65, 2.99) |
| P | 0.020 | 0.077 | 0.062 | 0.382 | 0.570 |

Linear regressions were also adjusted for eldest offspring status, mother's and father's education level and father's employment type. Observations in all analyses were weighted by the reciprocal of the number of siblings (of the specified sex and age) used in that analysis, and N_sw_ is the sum of weights for those whose fathers began smoking before 11 years old, followed by the total sum of weights. N_raw_ are the unweighted sample sizes. The analysis of all offspring ages was additionally adjusted for a cubic spline of offspring age. P_interaction_ tests whether the MD differs between sons and daughters.
